# Mental health impact of cuts to local government spending on cultural, environmental and planning services in England: a longitudinal ecological study

**DOI:** 10.1186/s12889-023-16340-0

**Published:** 2023-07-28

**Authors:** Katie Fahy, Alexandros Alexiou, Konstantinos Daras, Kate Mason, Davara Bennett, David Taylor-Robinson, Ben Barr

**Affiliations:** 1 Department of Public Health, Policy and Systems, University of Liverpool, Liverpool, L69 3GB UK; 2 Melbourne School of Population and Global Health, Centre for Health Policy, The University of Melbourne, Melbourne, Australia

**Keywords:** Mental health, Health inequality, Local government, Austerity, Culture, Environmental health, Planning

## Abstract

**Background:**

Over the past decade, there have been significant and unequal cuts to local authority (LA) budgets, across England. Cultural, environmental and planning (CEP) budgets have been cut by 17% between 2011 and 2019. This funding supports services such as parks, leisure centres, community development and libraries, all of which have potential to influence population mental health. We therefore investigated whether cuts to CEP services have affected mental health outcomes and the extent to which they have contributed to mental health inequalities between areas.

**Methods:**

Using fixed effects regression applied to longitudinal LA-level panel data in England, we assessed whether trends in CEP spend were associated with trends in mental health outcomes, between 2011 and 2019. The exposure was CEP spend and the primary outcome was the LA-average Small Area Mental Health Index (SAMHI). Additionally, we considered subcategories of CEP spend as secondary exposures, and antidepressant prescription rate and self-reported anxiety levels as secondary outcomes, both aggregated to LA-level. We adjusted all models for confounders and conducted subgroup analysis to examine differential mental health effects of spending cuts based on the level of area deprivation.

**Results:**

The average decrease in CEP spend of 15% over the period was associated with a 0.036 (95% CI: 0.005, 0.067) increase in SAMHI score, indicating worsening mental health. Amongst subcategories of CEP spending, cuts to planning and development services impacted mental health trends the most, with a 15% reduction in spend associated with a 0.018 (95% CI: 0.005, 0.031) increase in the SAMHI score. The association between cuts in CEP and deteriorating mental health was greater in more affluent areas.

**Conclusion:**

Cuts to spending on cultural, environmental, planning and development services were associated with worsening population mental health in England. Impacts were driven by cuts to planning and development services in particular. Reinvesting in these services may contribute to improved public mental health.

**Supplementary Information:**

The online version contains supplementary material available at 10.1186/s12889-023-16340-0.

## Introduction

There is growing evidence that public services, such as parks and libraries, are beneficial to public mental health, especially in less affluent areas where the costs associated with private services may be prohibitive. However, in England, these council-provided services, named cultural, environmental and planning (CEP) services, have been affected by austerity policies. Between 2011/12 and 2019/20, CEP service budgets were cut by 17%. These budget cuts have undoubtedly affected CEP service provision, particularly in more deprived areas where cuts were largest. These have potential implications for public mental health. In this study, we investigate how these geographically patterned changes in CEP expenditure have impacted mental health outcomes and geographical inequalities in mental health in England.

**Fig. 1:**
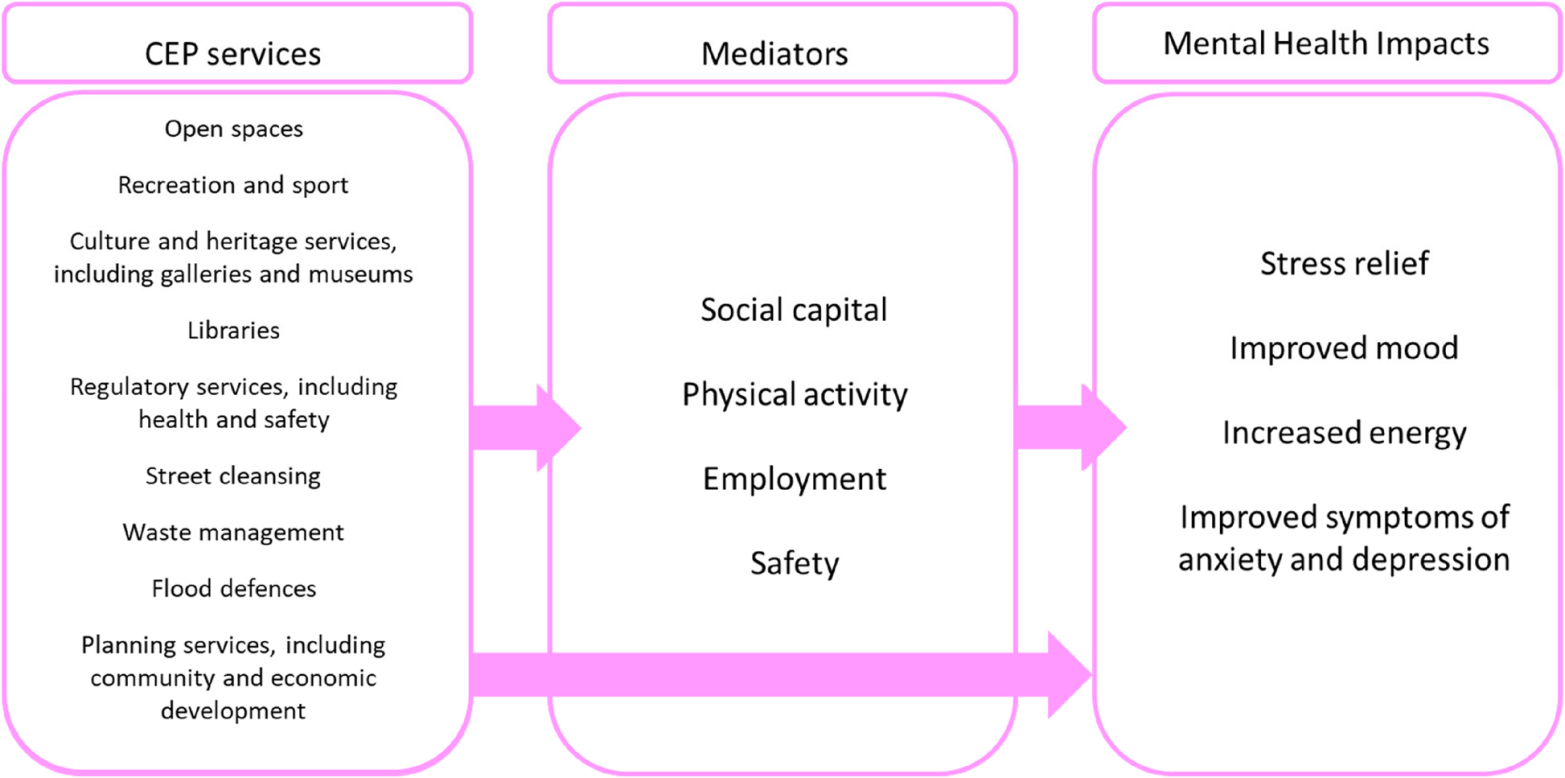
Logic model of pathways between CEP services and mental health impacts

There are a number of potential pathways from CEP services to mental health outcomes, as detailed in Fig. 1. CEP services such as libraries, parks, community development, museums and galleries enhance social cohesion of communities by providing space in which people may interact [1–4]. Greenspace has been found to improve perceptions of social capital and neighbourhood cohesion [2], and libraries promote social inclusion through activities including writing and painting groups, and providing designated safe spaces for vulnerable groups, such as LGBT youth [4]. Additionally, libraries provide safe, warm places for all, and are currently facing increasing demand due to the cost of living crisis [5, 6]. A key aim of planning services is to promote social interaction by improving connectivity of neighbourhoods and accessibility of social networks in the built environment [7–10]. Further, initiatives that engage the community in programmes such as neighbourhood regeneration and social inclusion have been evidenced to successfully improve social support and social inequalities [11, 12]. The mental health benefits of social cohesion have been well-documented [13–15].

Services such as parks and leisure centres may have a positive impact on population levels of physical activity [16, 17], with interventions such as park renovations and safety improvements increasing park usage and levels of physical activity [18]. Physical activity has been found to improve mental health outcomes [19–21] and there is some evidence that these benefits are enhanced when physical activity takes place in natural environments, such as parks [22]. Outdoor physical activity can be further facilitated by town planning services, through their influence on the built environment. Planning strategies may encourage active transport by introducing cycle lanes and improving walkability of neighbourhoods [7, 23, 24]. Through parks, leisure centres and planning services, local authorities hold influence over residents’ physical activity levels, and as such their resulting mental well-being.

As well as benefits mediated through physical activity, parks may affect mental health outcomes directly. Well-established psychological theories, including Ulrich’s Stress Reduction Theory [25] and Kaplan’s Attention Restoration Theory [26], highlight the mental health benefits of interacting with greenspace. Effects on stress reduction have been evidenced, including significant reductions in cortisol, an indicator of stress [3]. Systematic reviews have demonstrated the effects on attention restoration of exposure to green space, including the alleviation of hyperactivity and inattention problems in children [27], and some evidence of attention restoration for adults [28]. In addition, Kaplan theorised similar restorative effects of environments such as museums [29], which are also provided by local authorities through the CEP funding stream.

As outlined in the National Planning Policy Framework, planning services can improve economic growth and employment in the areas they serve, by setting an economic agenda, addressing potential barriers to development and supporting local services [10]. CEP funding also includes funding for training and employment initiatives [30]. Libraries have been evidenced to boost local economies, acting as tourist attractions [31],working in partnership with local jobcentres [32–34] or introducing schemes like Enterprising Libraries [35, 36], to improve local business and employment [4]. These impacts of CEP services on local employment levels and the economy have implications for mental health, as employment is recognised as one of the key social determinants of health [37] and has been evidenced to impact mental health in particular [38].

Furthermore, the digital inclusion libraries facilitate is beneficial for a range of other mediators that positively impact mental health, including education and social networks [31, 39, 40]. As highlighted in the COVID-19 pandemic, children from poorer backgrounds are often reliant on libraries’ digital access for schoolwork [41–44]. As well as access, libraries contribute to digital literacy needed to make the best use of services [45]. As education, work, welfare and social lives become increasingly digitalised, the role of libraries in bridging the digital divide becomes ever more important. Other mental health benefits of libraries relate to the provision of accessible reading materials [46]. Reading is strongly associated with mental wellbeing and life satisfaction [31, 47], and materials available include self-help books for conditions such as anxiety and depression, recommended by NICE as part of treatment [48–50], or those prescribed through schemes such as bibliotherapy [31]. Both digital inclusion and health literacy [4, 51–53] are key aims of the Universal Library Offer [54], with a focus on promoting equality to address the inverse care law [55, 56].

Community development strategies have been evidenced to be an effective approach to addressing root causes of poor physical and mental health in local communities [11, 12]. These area-based initiatives work with local communities to understand their needs and preferences, ensuring interventions are relevant and appropriate. NICE recommends these community-centred approaches and encourages engagement with those who are vulnerable or living in deprived areas especially, to address health inequalities [57].

Overall, CEP services potentially influence mental health through multiple determinants as defined by Barton and Grant [58], including natural and built environments, local economy, community, and lifestyle factors. Through these mediators and direct pathways, CEP services have the potential to influence the mental health outcomes of the communities they serve. The aim of this study is therefore to investigate whether areas with greater cuts to CEP services have experienced worse trends in mental health. We hypothesise that the reduction in local government funding in recent years that has disproportionately impacted CEP services may have negatively impacted the mental health and wellbeing of residents. We further hypothesise that any negative mental health impacts may be greater in more deprived areas where budget cuts have been more severe and residents may be more reliant on public services.

## Methods

### Setting

We conducted a longitudinal study at local authority level in England using panel data from 181 lower-tier local authorities between 2011 and 2019. In England, some areas have two tiers of local government: a county council (upper tier) and district councils (lower tier). Responsibilities are split between these councils, with CEP services being the main responsibility of district councils. In other areas of England, there is a single “unitary” level of local government which is responsible for all municipal services including social care. Previous studies have shown that unitary authorities tended to cut CEP budgets in order to protect social care services, which in themselves could impact on mental health [59]. These trade-offs between funding of service lines could lead to spurious associations between CEP spend and mental health in unitary authorities, potentially introducing biases to our study, therefore we excluded unitary authorities from our main analysis. Additionally, limiting analysis to lower-tier local authorities, i.e. district councils, provides a more homogenous group of areas to study and so unmeasured confounding will be minimised. Analysis including unitary authorities is presented in Appendix 5.

### Data

Our primary outcome was the Small Area Mental Health Index (SAMHI), a composite annual measure of population mental health for each Lower Super Output Area (LSOA) in England, available from the Place-Based Longitudinal Data Resource (PLDR) [60]. The SAMHI combines data on mental health outcomes from multiple sources into a single index. These include: mental health-related hospital attendances, prescription of antidepressants, percentage of adult patients with new diagnoses of depression and claimant rates of Incapacity Benefit or Employment Support Allowance for mental illness. The index has been computed for each year from 2011 to 2019. We combined data across LSOAs within each lower-tier local authority to obtain a measure of mental health at local authority level, weighting by LSOA population size. We then standardised the measure such that the mean of the index across all authorities and years is 0, with standard deviation of 1, and higher scores indicating worse mental health. We mapped all data to local authorities based on their 2021 boundaries.

We used self-reported anxiety levels as a secondary outcome, since this measure is not influenced by differing diagnostic practices across local authorities, as with SAMHI. Also, there is some evidence of a discrepancy between self-reported and objective measures of mental health, as objective measures are often dependent on accessing mental health services or treatment [61], so self-reported measures may be more representative [61]. Data is collected by the Office for National Statistics (ONS) on anxiety levels for each local authority through the Annual Population Survey (APS). For the well-being questions of the APS, including anxiety levels, there are approximately 150,000 respondents each year across the UK (382 local authorities); details on sampling are available in the quality and methodology information published by ONS [62, 63].Respondents are asked ‘Overall, how anxious did you feel yesterday?’, rated on a scale of 1 to 10, where 0 is ‘not at all’ and 10 is ‘completely’. We used the annual local authority average of survey responses to this question as provided by the ONS [64].

The primary exposure of interest was the gross expenditure per capita on CEP services. Gross expenditure includes spending by local authorities on provision of services and income raised in providing those services, for example, through fees and charges. The expenditure data is available at the lower-tier local authority level from the PLDR [65, 66]. To calculate this as a per capita measure, we used annual population estimates available from the Office for National Statistics. Expenditure data is available for financial years, i.e., from April 1^st^ to March 31^st^, such that 2011 refers to financial year 2011/2012. All expenditure data was adjusted for inflation using the consumer price index [67]. Details of the individual services included within CEP are available in the revenue outturn guidance documents [30]. In addition to overall expenditure, we considered the subcategories of cultural (arts, museums, theatres, parks, leisure facilities, libraries), environmental (trading standards, licencing, waste collection etc.), and planning and development services (planning, economic and community development) as secondary exposures to identify the mental health impact of each budget line.

**Fig. 2:**
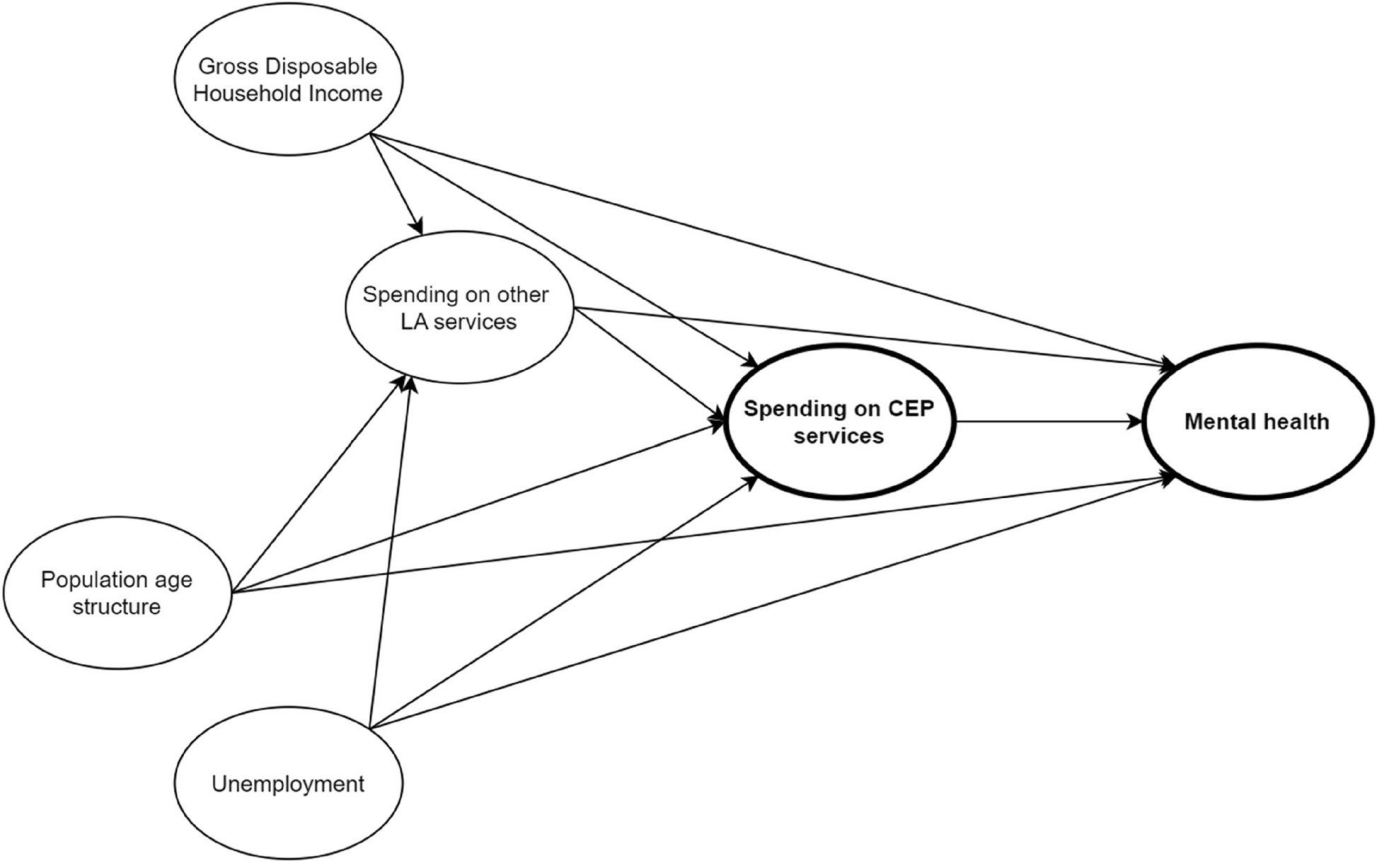
Directed Acyclic Graph of relationships between exposure, outcome and confounders

In all models, we controlled for additional time-varying place-based determinants of mental health outcomes, including levels of employment, income and age structure. We measured employment levels by the claimant rate for Job Seekers Allowance and, after 2013, Universal Credit [68]. We measured income levels using the Gross Disposable Household Income (GDHI) measured at lower-tier local authority level [69]. This measure can be interpreted as the income that households have available for spending or saving, after accounting for redistribution measures such as taxes and benefits [70]. Employment rates and income levels can also influence the funding received by local authorities as this is, in part, determined by local economic conditions. The age structure of a population also influences mental health outcomes and funding local authorities receive. We measured this using the proportion of the local authority population that is aged 65 and over. Therefore, each of these are potential confounders of the relationship between CEP spending and mental health outcomes. The hypothesised relationships are shown in the Directed Acyclic Graph (DAG) in Fig. 2, which represents the causal assumptions we are making to address our research question.

Further analysis investigated the differences in the relationship of CEP spend and mental health outcomes between areas with different levels of deprivation. We used the English Indices of Multiple Deprivation 2010 (IMD) as a measure of area deprivation in LSOAs. We assigned LSOAs to baseline deprivation quintiles, weighted by population size.

### Analysis

First, we graphically assessed the relationship between the change in CEP spending and the change in SAMHI – taking the difference of the values in 2011 and 2019 for each local authority. We repeated this for each of the three budget lines of CEP spend, i.e. cultural, environmental and planning and development services, to explore the relationship of each with SAMHI.

To assess how the trends in CEP spending have impacted mental health trends, we implemented fixed effects panel regression models for each outcome: SAMHI, rate of antidepressant prescriptions, and average anxiety levels, using annual data between 2011 and 2019 at the local authority level. We included fixed effects for local authority and year, and we used robust clustered standard errors to account for clustering within local authorities. We included gross CEP expenditure per capita as the exposure, and GDHI per capita, claimant rate, age structure and all other local authority spend per capita as covariates. We then repeated the models with each of cultural, environmental and planning and development services as the exposure, to assess which budget line has the biggest impact on mental health trends. We present the estimated change in outcome associated with a 15% decrease in funding for each exposure of interest – CEP, cultural, environmental or planning and development services. We chose to consider a 15% decrease in funding as this approximates the overall reduction in per capita spending on CEP over the study period. We include 95% confidence intervals for each estimate.

For the primary outcome of SAMHI, we considered the potential time lag between local authority spending on CEP services and the benefits to residents’ mental health. We repeated the fixed effects panel regression models with 1-, 2-, and 3-year time lags and calculated the AIC and BIC for each model to evaluate which best explained the relationship between CEP spend and mental health outcomes. In Appendix 1, we show that a model with 1-year lagged effects was the best fit, indicating that mental health outcomes can be best attributed to spending in the previous year. On the other hand, for the anxiety outcome, a model with no time lag provided the best fit according to AIC and BIC (Appendix 1). This may reflect self-reported anxiety levels being more responsive to changes in public services than the more objective measures included in SAMHI, such as diagnoses and prescriptions. Therefore, we present results from the 1-year lagged models, for all exposure-outcome combinations, except for analysis of anxiety levels which did not include lagged effects.

To assess inequalities in the mental health impacts of budget cuts, we conducted a subgroup analysis with SAMHI and covariates at the LSOA-level. As spend data is not available at LSOA-level, we assigned each LSOA spending figures of the LA they are part of. We fit models in subgroups of LSOAs according to the IMD quintile they belonged to. We fit similar models as previously, with panels defined as LSOAs and years, fixed effects for LSOAs and years, and standard errors clustered within LSOAs. Results of these models show us how the relationship between CEP spending and mental health varies between LSOAs with differing area deprivation. Subgroup analysis is our best approximation for this relationship, since data on expenditure at LSOA-level is not available.

### Robustness tests

To aid interpretation of the mental health impacts of budget cuts, we also analysed each component of the SAMHI separately. We present the rate of antidepressant prescriptions as a secondary outcome, as this overcomes the potential bias arising as both SAMHI and local authority resource allocation are partly determined by claimants of Incapacity Benefits or Employment Support Allowance. The rate of antidepressant prescriptions is measured in Average Daily Quantities (ADQ) per person, a unit developed to study variations in prescribing for important drug groups [71]. Results for the other components are presented in Appendix 4.

Analysis including unitary authorities, excluding London due to outlying trends in SAMHI [72], is presented in Appendix 5.

## Results

Table 1 summarises the mental health outcomes and local authority CEP spend at the start and end of the period that we studied, 2011 and 2019.

**Table 1:** Descriptive statistics for mental health outcomes and local authority CEP spend in 2011 and 2019

	**2011 (*n* = 181)**	**2019 (*n* = 181)**
	**Mean (s.d.)**	
**SAMHI**	-0.96 (0.55)	1.15 (0.85)
**Antidepressant rate (ADQ per capita)**	26.31 (4.32)	42.27 (7.56)
**Anxiety levels (1 – 10 scale)**	3.04 (0.31)	3.00 (0.38)^a^
**Total CEP spend (£ per capita)**	270.19 (50.33)	233.95 (44.23)
**Cultural spend (£ per capita)**	78.52 (26.99)	55.23 (24.58)
**Environmental spend (£ per capita)**	143.09 (33.8)	132.32 (23.67)
**Planning and development spend (£ per capita)**	48.58 (13.82)	46.40 (18.25)

^a^3 missing values

Across most measures, mental health worsened. The SAMHI increased from -0.96 in 2011 to 1.15 in 2019, an increase of 2.11 standard deviations. The rate of prescriptions of antidepressants increased from 26.31 ADQ per capita in 2011, to 42.27 ADQ per capita in 2019. However, on average, people reported slightly lower levels of anxiety, decreasing from 3.04 to 3.00 (out of 10) over the period.

Annual local authority spending per capita on all CEP services decreased from £270 to £234 over the period. In both absolute and relative terms, cultural services experienced the largest budget cuts of £23 per capita, or 30%, between 2011 and 2019.

Descriptive statistics for the other components of SAMHI are presented in Appendix 2. Appendix 3 shows spaghetti plots of the trends in CEP spending,SAMHI and anxiety throughout the period, including trends by level of deprivation. SAMHI has steadily increased over the study period, with larger increases in more deprived areas, whereas anxiety scores have remained relatively stable with a less clear deprivation gradient. Cuts to CEP spending were made incrementally throughout the period.

**Fig. 3:**
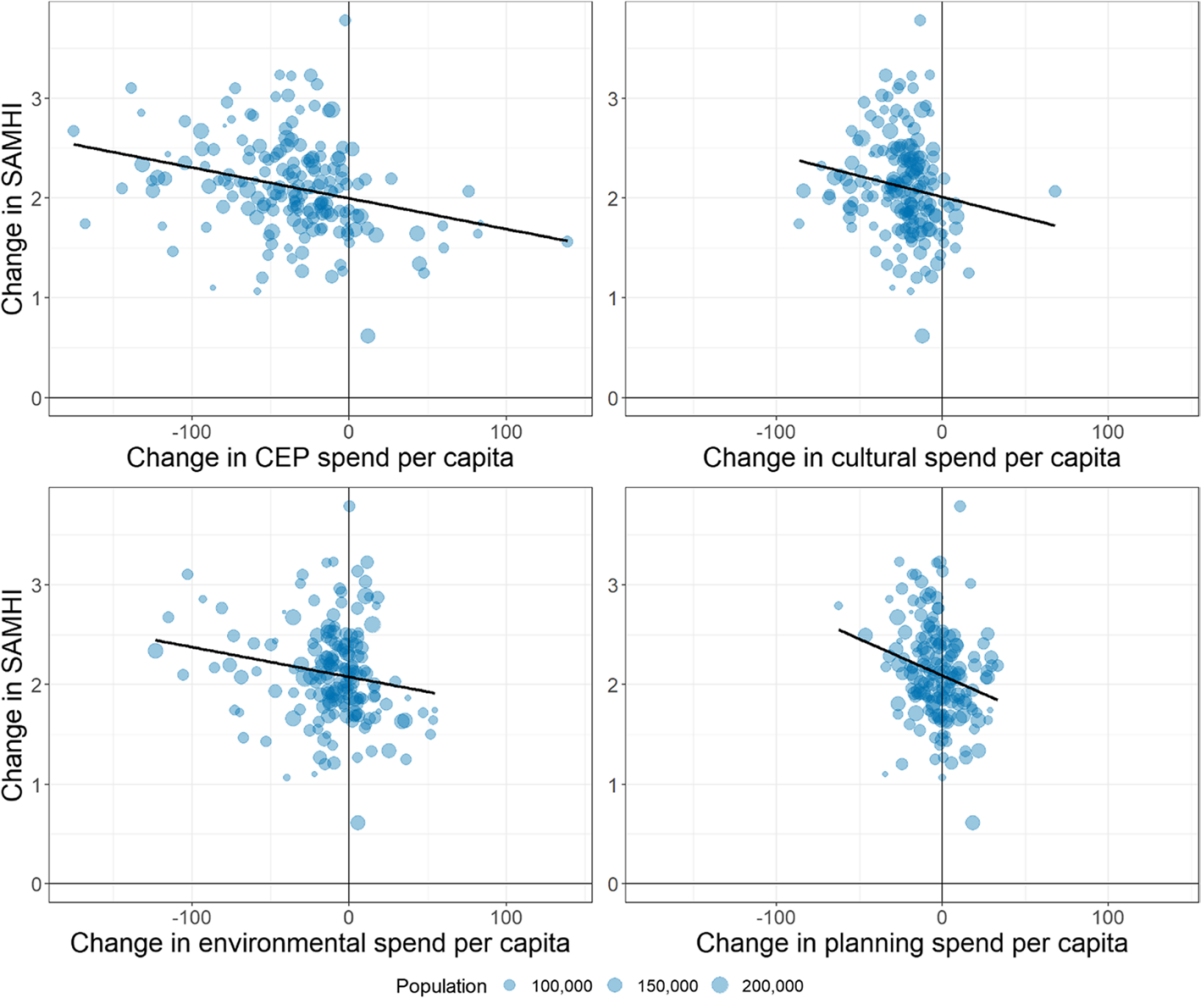
Change in CEP spend per capita against change in SAMHI, between 2011 and 2019 by local authority

Figure 3 shows the change in total CEP spend per capita against the change in SAMHI for each local authority over the study period, including a breakdown for each budget line. The size of each bubble reflects the local authority population size in 2019. Overall, local authorities with the largest CEP budget cuts experienced the biggest increase in SAMHI, indicating worsening mental health in those areas. This trend is especially evident for planning and development services, for which there is the steepest decline in mental health as budget cuts increase.

Table 2 presents the change in each mental health outcome for a 15% decrease in CEP spending, estimated by the fixed effects panel regression models. Overall, the models show some association between CEP spending and mental health, with most estimated effects in the hypothesised direction.

**Table 2:** Results of adjusted fixed effects panel regression models of trends in mental health outcomes by trends in local authority CEP spending. Estimates presented are the estimated change in mental health outcome for a 15% decrease in spending, with 95% confidence intervals

	**SAMHI**^**a**^	**Antidepressant rate**^**b**^	**Anxiety levels**^**c**^
	**Change in outcome for 15% decrease in spending (95% CI)**		
Total CEP	0.036 (0.005, 0.067)	0.13 (-0.09, 0.36)	0.035 (0.003, 0.066)
Cultural	-0.005 (-0.022, 0.013)	0.01 (-0.14, 0.16)	0.014 (-0.006, 0.034)
Environmental	0.019 (-0.013, 0.05)	-0.09 (-0.28, 0.11)	0.007 (-0.021, 0.034)
Planning	0.018 (0.005, 0.031)	0.13 (0.04, 0.22)	0.018 (0.001, 0.036)

^a^Standard deviation change, positive effect sizes indicate worsening mental health

^b^ADQ per capita change, positive effect sizes indicate more antidepressants prescribed

^c^Change in rating of anxiety out of 10, positive effect sizes indicate higher anxiety. Lagged effects are not included in analysis of anxiety, and study period limited to 2011 to 2018 due to data availability

For the primary outcome, a 15% decrease in total CEP spending was associated with a 0.036 (95% CI: 0.005, 0.067) standard deviation increase in SAMHI. Cuts to planning services were associated with worsening SAMHI scores whereas the effects of cultural and environmental cuts were negligible. The estimated effect of planning spending cuts was statistically significant, with a 15% decrease in spending associated with a 0.018 (95% CI: 0.005, 0.031) standard deviation increase in SAMHI.

Similarly, a 15% decrease in total CEP spending was associated with increasing rates of antidepressant prescriptions, with an estimated 0.13 (95% CI: -0.09, 0.36) change in ADQs prescribed per person. Effects of the cultural and environmental budget lines on antidepressant prescriptions were negligible, though a 15% planning budget cut was associated with a significant 0.13 (95% CI: 0.04, 0.22) increase in ADQs prescribed per person.

We estimate a small increase in anxiety levels of 0.035 (95% CI: 0.003, 0.066) for a 15% decrease in total CEP spending. Planning services are the only budget line for which a 15% spending cut was significantly associated with worsening anxiety levels, with an estimated 0.018 (95% CI: 0.001, 0.036) increase in rating (out of 10).

Results for the other components of SAMHI are presented in Appendix 4, providing some additional evidence of an association between CEP spending and mental health outcomes, in particular new diagnoses of depression per capita, which increased by 0.10 (95% CI: 0.003, 0.204) with a 15% cut in total CEP spend. When investigating specific budget lines, we found a 15% cut in environmental spending was associated with a 0.10 (95% CI: 0.002, 0.21) increase in new diagnoses of depression per capita. We also found associations between planning budget cuts and increases in mental health-related hospital attendances. These increases were small with 95% confidence intervals that exclude zero. Effects of cultural budget cuts were negligible, and we found no association between the budget cuts and claimants of mental health-related unemployment benefits. Results for the analysis including unitary authorities (Appendix 5) show smaller estimated effects and the effect of CEP cuts on anxiety levels is negligible. Further, when we include London authorities, the estimated effects to SAMHI are smaller and negligible for the total CEP spend.

Table 3 presents the results of models comparing the mental health impacts of CEP spending in LSOAs with different levels of deprivation, as measured by IMD. Decreases in total CEP spend were associated with worsening mental health in all but the most deprived areas. Across all types of CEP spending, the least deprived quintile of LSOAs experienced small significant increases in SAMHI as spending reduced, indicating worsening mental health. For example, a 15% reduction in total CEP spending was associated with a 0.028 (95% CI: 0.020, 0.037) standard deviation increase in SAMHI for the least deprived LSOAs. On the other hand, in the most deprived LSOAs there was no association between CEP spend and mental health trends, with SAMHI changing by -0.013 (95% CI: -0.028, 0.001) standard deviations as total CEP spending decreases by 15%. Only cuts to planning spending were associated with worsening mental health across all quintiles – though the estimated effect was smallest in the most deprived areas, at 0.008 (95% CI: 0.002, 0.013) standard deviations compared to 0.011 (95% CI: 0.007, 0.014) standard deviations in the least deprived LSOAs.

**Table 3:** Results of adjusted fixed effects panel regression models of trends in SAMHI by trends in local authority CEP spending, comparing effects of spend between IMD quintiles of LSOAs

	**Change in SAMHI**^**a**^** for 15% decrease in spending (95% CI)**			
	Total CEP	Cultural	Environmental	Planning
All LSOAs	0.027 (0.022, 0.032)	0.006 (0.003, 0.008)	0.009 (0.005, 0.014)	0.014 (0.012, 0.016)
Least deprived LSOAs	0.028 (0.02, 0.037)	0.008 (0.003, 0.013)	0.023 (0.015, 0.031)	0.011 (0.007, 0.014)
2nd quintile	0.017 (0.008, 0.026)	0.005 (-0.001, 0.01)	0.008 (-0.001, 0.016)	0.01 (0.006, 0.014)
3rd quintile	0.031 (0.021, 0.041)	0.001 (-0.005, 0.007)	0.015 (0.006, 0.025)	0.016 (0.011, 0.02)
4th quintile	0.03 (0.02, 0.04)	0.008 (0.002, 0.014)	0.009 (0, 0.019)	0.011 (0.007, 0.016)
Most deprived LSOAs	-0.013 (-0.028, 0.001)	0.003 (-0.005, 0.011)	-0.026 (-0.038, -0.014)	0.008 (0.002, 0.013)

^a^Standard deviation change, positive effect sizes indicate worsening mental health. Estimates are adjusted for GDHI per capita, claimant rate, area age structure and other LA spending

## Discussion

### Summary of findings

This study provides evidence that cuts to local government spending on cultural, environmental and planning services may have harmed public mental health, though estimated effects were small. These mental health impacts appear to be driven primarily by cuts to the planning and development budgets, which cover the councils’ community and economic development services as well as more traditional planning activities. When investigating inequalities in trends, we found a greater association between cuts and deteriorating mental health in more affluent areas compared to more deprived areas. Our study indicates that it is likely the austerity policy implemented in 2010 contributed to a deterioration in mental health.

### Results in the context of past research

There is widespread evidence of the negative public health effects of the local authority budget cuts implemented in England since 2010. For example, stalling life expectancy improvements [73], rising drug-related deaths [74], and rising A&E admissions [75], have all been attributed to the cuts to public services. Our study adds to this evidence base, showing that CEP budget cuts may have contributed to deteriorating mental health. On average CEP budgets were cut by 15%, which was associated with a 0.036 (95% CI: 0.005, 0.067) standard deviation increase in the SAMHI. To contextualise this effect size, it is 1.7% of the overall 2.11 standard deviation increase (worsening) in the SAMHI between 2011/12 and 2019/20. This is a small but significant proportion given the number of other factors that have negatively impacted mental health over the period, such as falling incomes, increasing unemployment and changes to the welfare system [76, 77]. This finding was supported by our analysis of secondary outcomes, which showed increasing antidepressant prescriptions and anxiety levels associated with budget cuts.

As discussed in the introduction, previous research has shown the mental health benefits of CEP services. These include benefits of libraries, museums, parks, environmental health, economic and community development projects to social cohesion [8, 9], reading [31, 47, 53], interacting with nature [26], digital access [39, 40, 44, 56] and physical activity [16, 17, 22]. All of which ultimately support residents’ mental health and well-being [1, 13, 14, 19, 20]. Therefore, with budget cuts resulting in 800 closed libraries in the last decade [78], numerous closed leisure centres [79] and overall reduced CEP service availability and maintenance [80], it seems plausible that population mental health would be impacted. Our research corroborates this theory, providing some evidence of the contribution of CEP budget cuts to deteriorating mental health.

When considering specific budget lines within CEP services, we found that planning and development services had been especially influential to public mental health. This aligns with previous research on the benefits of planning services, particularly economic and community development which have been cut significantly [81]. For example, the recent Communities in Control study of investments in community empowerment initiatives resulting from the Big Local programme found benefits to well-being, as measured by SAMHI and anxiety levels from the Annual Population Survey [82]. Similarly, a study of economic development in Preston found significant benefit to mental health, as measured by SAMHI [83]. Our study of the same outcomes supports this evidence, as well as evidencing small impacts on antidepressant prescription rates and mental health-related hospital attendances resulting specifically from cuts to planning and development services.

On the other hand, we found cuts to cultural and environmental services had a negligible negative effect on mental health, with the exception of cuts to environmental services significantly increasing diagnoses of depression. This contradicts previous research on the mental health benefits of cultural and environmental services, especially the wide evidence base for libraries [4, 31, 53]. This may be due to barriers to accessing services, such as cost, travel or social barriers [84, 85]. Accessibility issues may prevent those at risk of poor mental health from experiencing the benefits of cultural services.

We found a greater association between cuts and deteriorating mental health in more affluent areas compared to more deprived areas. This runs counter to our original hypothesis. Cuts have been inequitable, with more deprived areas of England experiencing steeper budget cuts, and cuts for CEP services being much larger than other local authority services [59]. As such, we hypothesised that mental health in more deprived areas would be most impacted by budget cuts. However, our results contradict this and instead corroborate previous research identifying public cultural services such as parks and museums as so called ‘pro-rich’ services [86]. Our counterintuitive findings may be indicative of the barriers to accessing CEP services in more deprived areas, including cost and distribution of services [87–89]. There has also been evidence of lower quality services in poorer areas [90, 91], which could potentially contribute to weaker associations with mental health. Alternatively, our findings could be evidence of ecological fallacy arising from studying area-level deprivation and aggregated individual mental health outcomes. In particular, recent research has shown that the majority of socioeconomically deprived individuals do not live in the most deprived areas, as measured by IMD [92]. As such, it may be the most disadvantaged residents of affluent areas that have been impacted by CEP budget cuts, accounting for the greater association we found in these areas.

Moreover, the mental health impacts may vary across services dependent on the way budget cuts have been implemented and public response to cuts. In many places, budget cuts have resulted in neo-liberalisation of services such as parks and libraries, diminishing their accessibility [93–95]. However, in some places, local community groups have responded by taking over management of services [96] or successfully contesting proposals for privatisation [97]. As well as protecting services from commercialisation or reduced quality of services, there is some evidence that this localisation has been beneficial, allowing services to be more responsive to community needs [98]. However, the financial and social capital that enables such volunteer groups to operate is unevenly distributed across the country [98]. The ability to organise volunteer groups may also vary across urban and rural areas. Others argue that underfunding of parks and declining public interest in libraries precede austerity [96, 99]. These factors could explain why we found no association between cultural budget cuts and mental health outcomes.

### Strengths and limitations

The main strength of our study is the use of longitudinal data capturing a period of changing mental health outcomes and spending on CEP services. This allowed us to study the relationship between mental health and spending over time using fixed effects approaches to account for time-invariant differences between local authorities. The Place-based Longitudinal Data Resource provides consistent and comparable time-series that account for different LA types and changes in LA administrative geography [100]. However, we could not account for potential bias introduced by measurement error in the local government spending data, or differences in reporting spending between places and over time. Additionally, previous research has shown fixed effects approaches may result in conservative estimates, especially when clustering whole population data rather than a sample, as in our study [101]. Further, though we account for confounding by the time-varying factors of population age structure, unemployment rates and local incomes, it is possible that there are unobserved time-varying factors for which we were not able to control. This could include, for example, budget lines that are not controlled at LA-level.

Furthermore, this is the first study of the mental health impacts of CEP budget cuts, to our knowledge, with only one previous study of public health impacts investigating childhood obesity [102]. CEP services are often overlooked, despite the benefits they provide to public health and well-being. They have been notably deprioritised in recent years as local authorities have been forced to make cuts to non-statutory services. However, the long-term health impacts of these budget cuts are not yet known, so our study is an important first step in providing this evidence.

One limitation of our study is that the primary outcome, SAMHI, is determined in part by trends in prescribing and diagnosing, which may vary between local authorities [103, 104]. To overcome this limitation, we have considered secondary outcomes not affected by this, i.e. self-reported anxiety levels, mental health-related hospital attendances and claimants of Incapacity Benefits or Employment Support Allowance for mental health reasons. These secondary outcomes show similar results.

In addition, the data we use to measure antidepressant prescriptions only includes those prescribed by GPs. So, for example, antidepressants prescribed in a hospital would not be included. However, this is unlikely to significantly affect our study as the pathways between CEP services and mental health outcomes are less likely to include severe mental illness treated outside of GP practices. Again, we studied secondary outcomes excluding antidepressant prescriptions and found similar results.

Our study is also limited by the time period considered. Including data on CEP spending and mental health outcomes prior to 2011 would enable comparisons of trends before and after the introduction of austerity. However, the SAMHI and measure of anxiety from the Annual Population Survey are only available from 2011 onwards. Previous research has found that use of antidepressants was increasing prior to 2011, but the rate accelerated from 2008 with the financial crisis and continued to increase at this greater rate during the period of austerity [103, 105].

Further, our study is limited by the use of area-level data, which prevents us from accounting for or assessing individual differences in impacts on the basis of major determinants of mental health, such as age, sex and ethnicity [106, 107]. This may introduce bias through the ecological fallacy as it is likely that individuals within local authorities will have been differently affected by the CEP budget cuts [108]. Similarly, aggregating data to local authority level limits our ability to assess variance in mental health outcomes within local authorities. Data on local expenditure at neighbourhood level is not available and, in many cases, it would not be possible or relevant to estimate. CEP spending within an LA will in practice happen at multiple geographies, such as central libraries, leisure centres, and local community centres.

## Conclusions and implications for policy and research

In this study, we have found that the budget cuts to CEP services have had a small but significant impact on mental health in England, with cuts linked to more people experiencing poor mental health. This is important as health impacts of CEP service budget cuts have not previously been explored, and are often minimised in relation to cuts to other public services. Our findings are important in the current context of increasing mental health problems since the COVID-19 pandemic, as well as the additional pressures that CEP services will be experiencing due to the cost of living crisis, including increasing running costs and demand as individuals use public spaces to cut their home energy costs [5]. Therefore, it’s vital that the government reinvest in CEP services, particularly planning and community development, as part of their Mental Health Recovery Plan [109]. Policies to support public mental health and well-being are needed now more than ever.

## Supplementary Information

**Additional file 1.**

## Data Availability

The datasets used and/or analysed during the current study are available from https://pldr.org/ and further guidance for the data can be forwarded to the corresponding author on reasonable request.

## Abbreviations

CEP: Cultural, environmental and planning
LA: Local authority
UK: United Kingdom
IMD: Index of Multiple Deprivation
CPI: Consumer Price Index
