# Supplementary material for: Mental health impact of cuts to local government spending on cultural, environmental and planning services in England: a longitudinal ecological study
Source: BMC Public Health. 2023 Jul 28;23:1441. doi: 10.1186/s12889-023-16340-0 (PMC10375661; doi:10.1186/s12889-023-16340-0)
Supplement: Supplementary file 1 — Additional file 1. [file 12889_2023_16340_MOESM1_ESM.pdf]

Appendix 1

Results of adjusted fixed effects panel regression models of trends in SAMH and anxiety levels, comparing AIC and BIC for lagged effects on mental health.

	**Change in outcome for 15% decrease in CEP spending**	**AIC**	**BIC**
**Outcome - SAMHI**			
**No time lag**	0.035 (0.004, 0.065)	-649.14	369.37
**1-year time lag**	0.027 (-0.002, 0.056)	-651.95	366.43
**2-year time lag**	0.007 (-0.022, 0.037)	-633.53	384.97
**3-year time lag**	0.007 (-0.027, 0.041)	-611.08	407.43
**Outcome – anxiety level**			
**No time lag**	0.035 (0.004, 0.066)	885.91	1904.15
**1-year time lag**	-0.028 (-0.057, 0.001)	892.40	1910.51
**2-year time lag**	-0.016 (-0.046, 0.014)	894.65	1912.89
**3-year time lag**	-0.008 (-0.044, 0.027)	894.58	1912.82

Appendix 2

Descriptive statistics for components of SAMHI in 2011 and 2019.

	**Mean (s.d.)**	
	**2011**	**2019**
**Depression diagnosis**	6.04 (1.17)	11.28 (1.86)
**Incapacity benefits**	2.20 (0.77)	1.99 (0.69)
**Mental health-related hospital attendances (z-scores)**	-0.96 (0.61)	0.99 (0.91)

Depression diagnosis measured as % of population with a new diagnosis in previous year. Mental health-related hospital attendances measured as number of attendances per person. Incapacity benefits measured as percent of people age 16-64 claiming incapacity benefit and employment support allowance for mental illness.

Appendix 3

Plot of CEP spend between 2011 and 2019 in lower-tier local authorities.

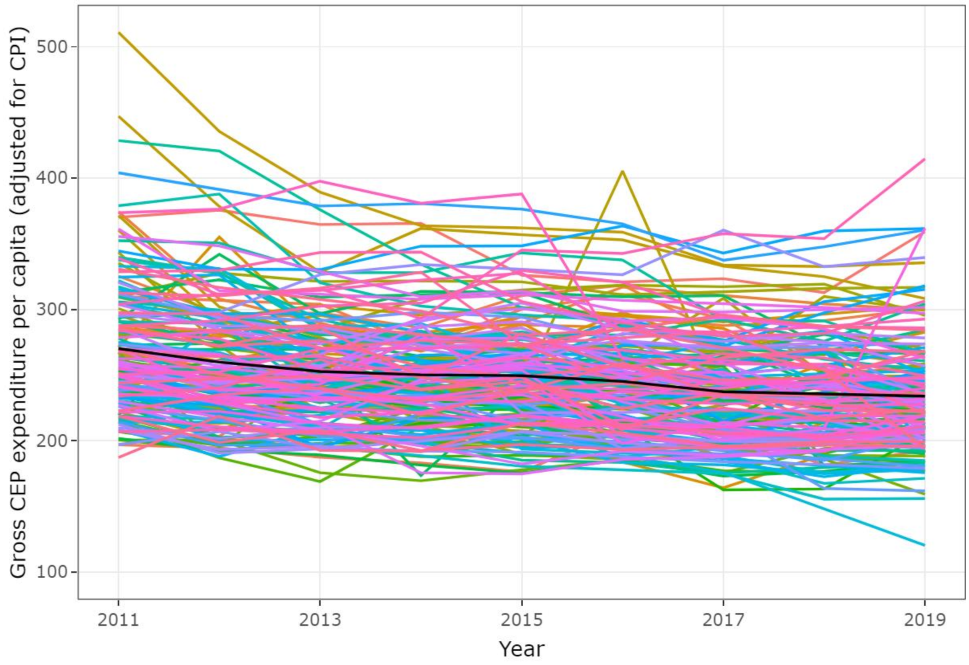

Plot of average SAMHI between 2011 and 2019 in lower-tier local authorities.

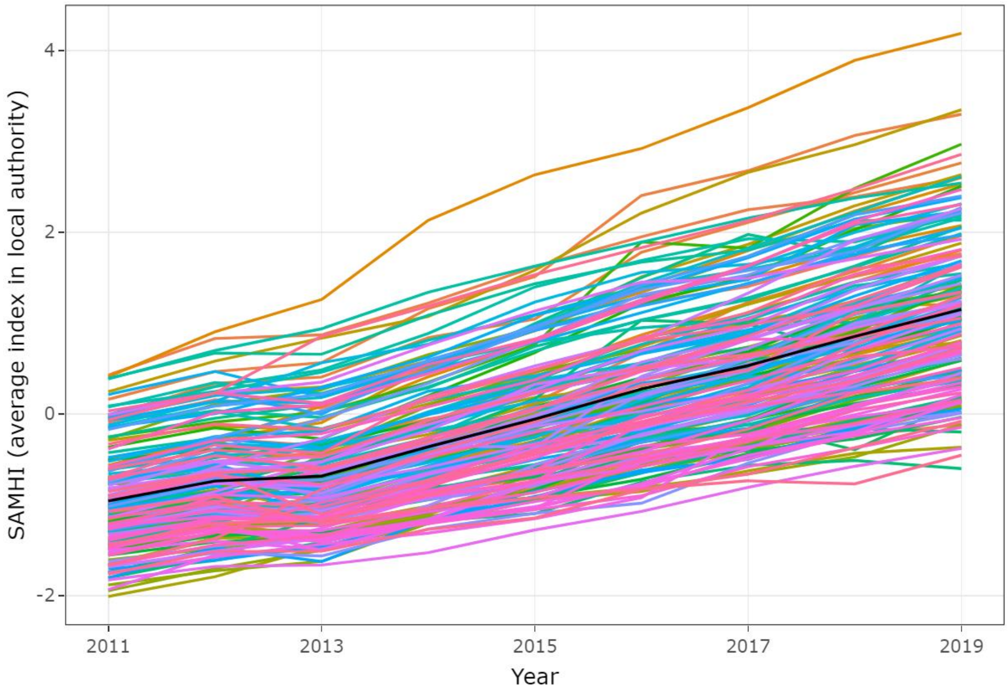

Plot of average SAMHI between 2011 and 2019 in lower-tier local authorities.

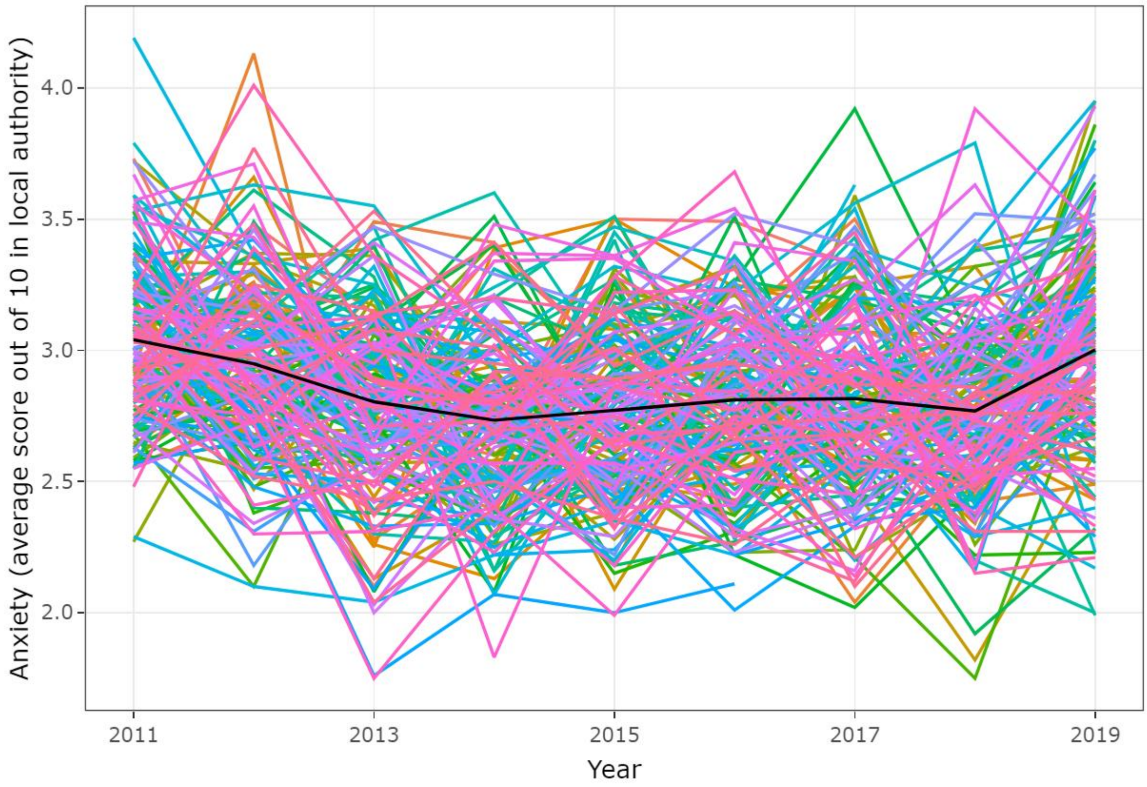

Plot of average CEP spend between 2011 and 2019 in lower-tier local authorities, stratified by IMD quintiles.

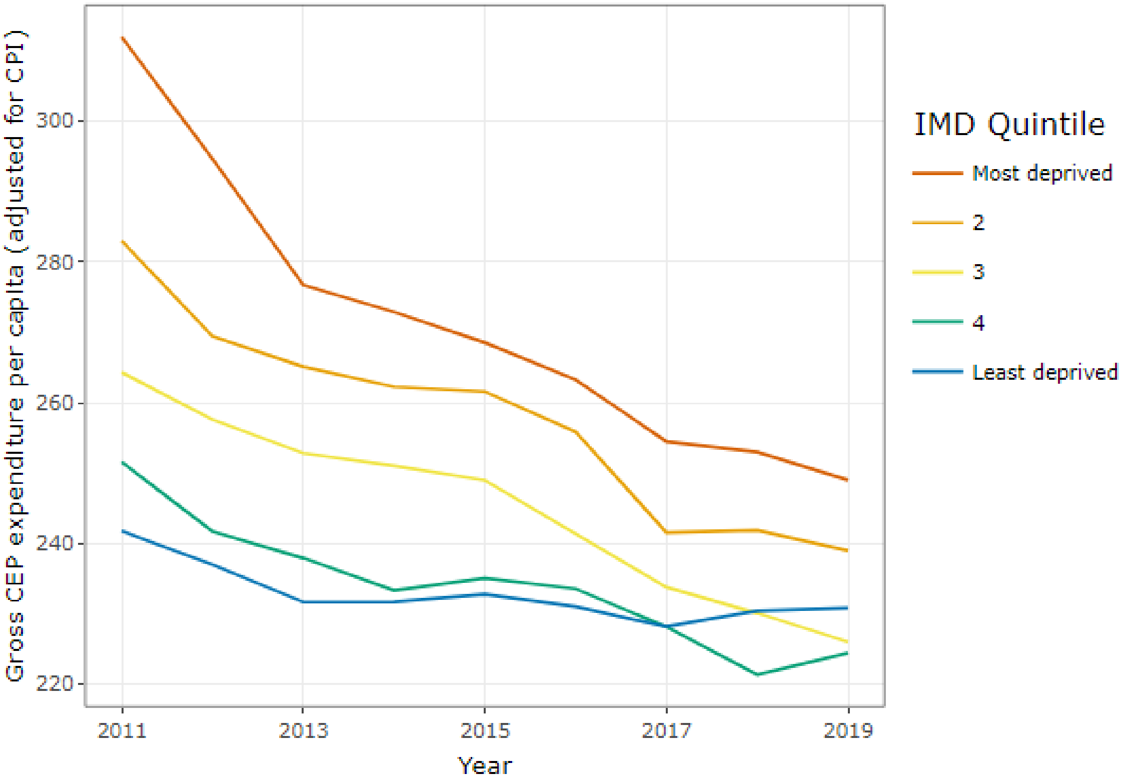

Plot of average SAMHI between 2011 and 2019 in lower-tier local authorities, stratified by IMD quintiles.

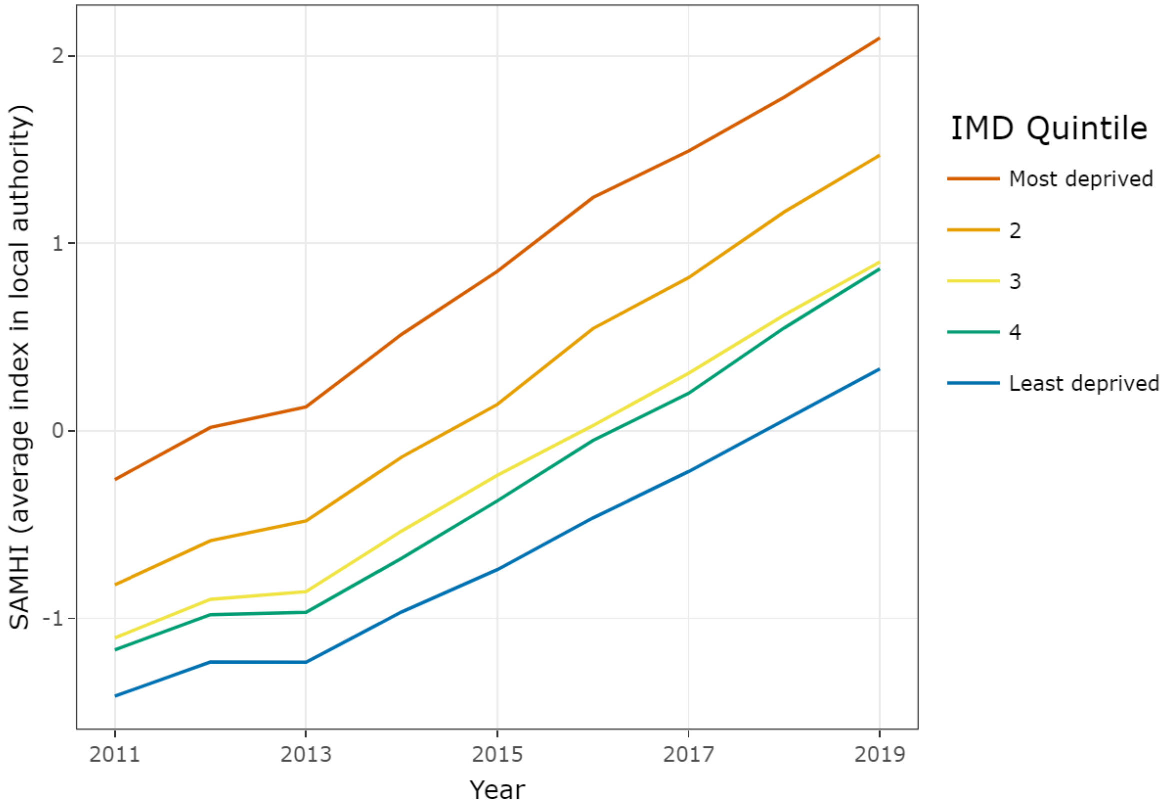

Plot of average anxiety between 2011 and 2019 in lower-tier local authorities, stratified by IMD quintiles.

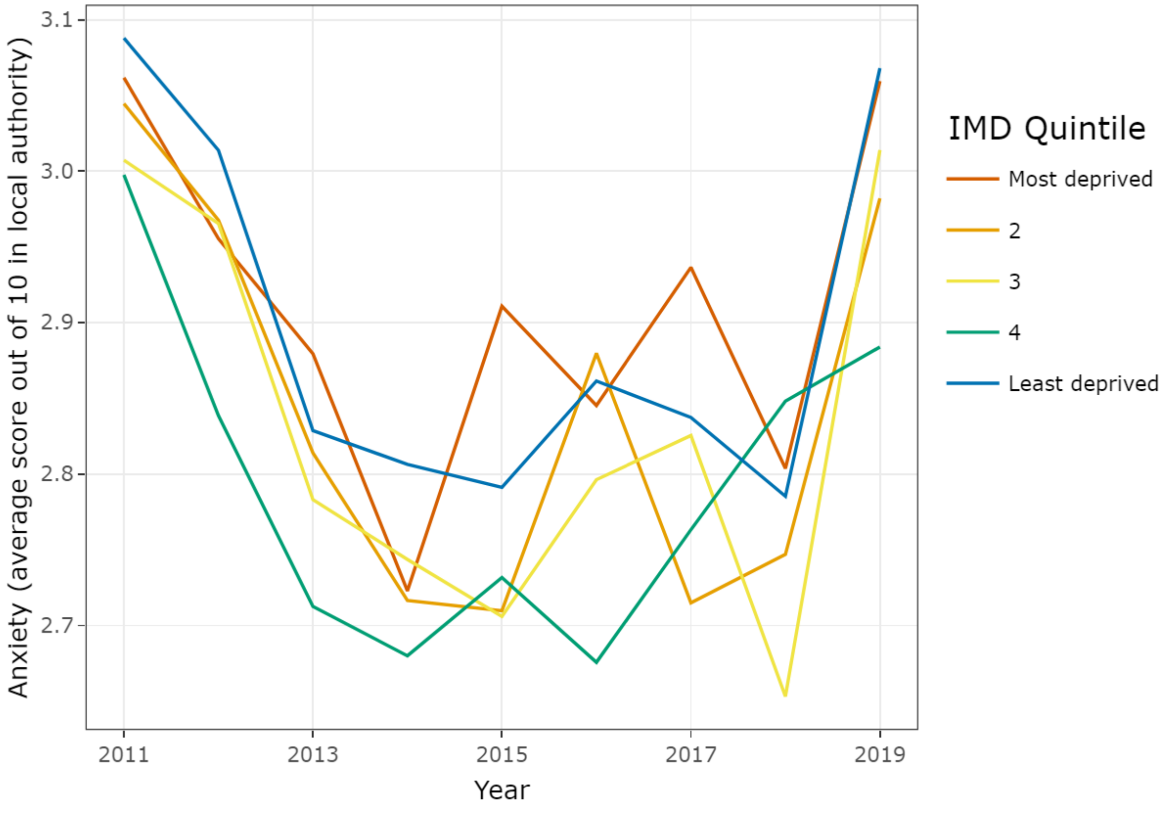

Appendix 4

Results of adjusted fixed effects panel regression models of components of SAMHI.

	Depression diagnosis	Incapacity benefits*	Mental health-related hospital attendances
	**Change in outcome for 15% decrease in spending (95% CI)**		
CEP	0.103 (0.003, 0.204)	0.01 (-0.014, 0.034)	0.015 (-0.029, 0.059)
Cultural	-0.028 (-0.083, 0.028)	0.007 (-0.007, 0.021)	0.003 (-0.024, 0.03)
Environmental	0.104 (0.002, 0.206)	0.005 (-0.015, 0.025)	-0.023 (-0.064, 0.018)
Planning	0.037 (-0.003, 0.076)	-0.001 (-0.009, 0.007)	0.022 (0.001, 0.043)

Depression diagnosis measured as % of population with a new diagnosis in previous year. Mental health-related hospital attendances measured as number of attendances per person. Incapacity benefits measured as percent of people age 16-64 claiming incapacity benefit and employment support allowance for mental illness.
*Estimates for incapacity benefits adjusted for other LA spending only. GDHI and Job Seeker's allowance/Universal Credit claimant rate not relevant confounders for this outcome.

Appendix 5

Results of adjusted fixed effects panel regression models including lower-tier local authorities, unitary authorities and London boroughs.

	SAMHI[a]	Antidepressant rate[b]	Anxiety levels[c]
	**Change in outcome for 15% decrease in spending (95% CI)**		
All LAs excluding London (274 LAs, 9 years)			
CEP	0.028 (0.001, 0.054)	0.1 (-0.08, 0.28)	0.018 (-0.001, 0.037)
Cultural	-0.006 (-0.016, 0.004)	-0.02 (-0.1, 0.06)	0.004 (-0.005, 0.013)
Environmental	0.013 (-0.007, 0.033)	-0.05 (-0.2, 0.1)	0.004 (-0.012, 0.021)
Planning	0.016 (0.006, 0.026)	0.1 (0.03, 0.17)	0.011 (0, 0.021)
All LAs including London (288 LAs, 9 years)			
CEP	0.014 (-0.011, 0.039)	0.12 (-0.05, 0.29)	0.018 (0, 0.037)
Cultural	-0.011 (-0.021, -0.001)	-0.07 (-0.14, 0.01)	0.003 (-0.006, 0.012)
Environmental	0.006 (-0.012, 0.024)	0 (-0.15, 0.14)	0.006 (-0.01, 0.021)
Planning	0.012 (0.003, 0.021)	0.1 (0.03, 0.16)	0.011 (0.001, 0.020)

[a] Standard deviation change, positive effect sizes indicate worsening mental health
[b] ADQ per capita change, positive effect sizes indicate more antidepressants prescribed
[c] Change in rating of anxiety out of 10, positive effect sizes indicate higher anxiety
